# Thyroid Hürthle Cell Carcinoma: Clinical, Pathological, and Molecular Features

**DOI:** 10.3390/cancers13010026

**Published:** 2020-12-23

**Authors:** Shoko Kure, Ryuji Ohashi

**Affiliations:** Integrated Diagnostic Pathology, Nippon Medical School, 1-1-5 Sendagi, Bunkyoku, Tokyo 113-8602, Japan; r-ohashi@nms.ac.jp

**Keywords:** Hürthle cell carcinoma, thyroid cancer, oncocytic

## Abstract

**Simple Summary:**

Hürthle cell carcinoma (HCC) represents 3–4% of thyroid carcinoma cases. It is characterized by its large, granular and eosinophilic cytoplasm, due to an excessive number of mitochondria. Hürthle cells can be identified only after fine needle aspiration cytology biopsy or by histological diagnosis after the surgical operation. Published studies on HCC indicate its putative high aggressiveness. In this article, current knowledge of HCC focusing on clinical features, cytopathological features, genetic changes, as well as pitfalls in diagnosis are reviewed in order to improve clinical management.

**Abstract:**

Hürthle cell carcinoma (HCC) represents 3–4% of thyroid carcinoma cases. It is considered to be more aggressive than non-oncocytic thyroid carcinomas. However, due to its rarity, the pathological characteristics and biological behavior of HCC remain to be elucidated. The Hürthle cell is characterized cytologically as a large cell with abundant eosinophilic, granular cytoplasm, and a large hyperchromatic nucleus with a prominent nucleolus. Cytoplasmic granularity is due to the presence of numerous mitochondria. These mitochondria display packed stacking cristae and are arranged in the center. HCC is more often observed in females in their 50–60s. Preoperative diagnosis is challenging, but indicators of malignancy are male, older age, tumor size > 4 cm, a solid nodule with an irregular border, or the presence of psammoma calcifications according to ultrasound. Thyroid lobectomy alone is sufficient treatment for small, unifocal, intrathyroidal carcinomas, or clinically detectable cervical nodal metastases, but total thyroidectomy is recommended for tumors larger than 4 cm. The effectiveness of radioactive iodine is still debated. Molecular changes involve cellular signaling pathways and mitochondria-related DNA. Current knowledge of Hürthle cell carcinoma, including clinical, pathological, and molecular features, with the aim of improving clinical management, is reviewed.

## 1. Introduction

Oncocytic tumors are found in various organs, including salivary glands, lacrimal glands, pancreas, liver, kidney, thyroid, parathyroid and pituitary glands. Thyroid neoplasms composed of oncocytic cells are called Hürthle cell tumors. These are also known as oncocytic/oxyphilic follicular cell tumors and used to be classified as a variant of follicular thyroid tumors. In the latest WHO classification [1], Hürthle cell tumors are identified as a special type of tumor derived from thyroid follicles, and distinguished from thyroid follicular tumors. Hürthle cell tumors can be benign or malignant. Hürthle cell tumors with capsular and/or vascular invasion, lymph nodes metastasis, or distant metastasis are Hürthle cell carcinoma (HCC). HCC represents 3–4% of thyroid carcinoma cases [2,3]. HCC has been considered as a more aggressive form of carcinoma compared to non-oncocytic thyroid carcinomas. Due to its rarity and conflicting information from previous studies, the pathological characteristics and biological behavior of HCC remain to be elucidated. In fact, most studies reporting on the biological features of thyroid differentiated tumors have included HCC as a part of follicular thyroid cancer or have failed to adequately separate malignant Hürthle cell tumor from benign tumor. Thus, studies especially focusing only on pure HCC are few [4]. There is still no consensus on the optimal treatment method for HCC, and the postoperative effect of radioactive iodine treatment is unclear. In this article, current knowledge of HCC, including clinical, pathological, and molecular features, is reviewed, with the aim of improving clinical management.

## 2. Nomenclature and Definition

Historically, the name “Hürthle” was first reported in 1894 by Karl Hürthle. Originally, it was used to refer to an ultimobranchial body derived from C cells (parafollicular cells) in the thyroid of dogs. Then, in 1898, Max Ashkanazy first described oncocytic follicular-derived cells in patients with Graves’ disease as the current Hürthle cells (Ashkanazy cells). The term Hürthle cell has been appended to the cells described by Ashkanazy, and the name has continued to be used until today [5]. 

Hürthle cells are characterized cytologically as large cells with abundant eosinophilic, granular cytoplasms, and large hyperchromatic nuclei with prominent nucleoli. The cytoplasm of a Hürthle cell is swollen due mainly to the presence of numerous mitochondria. The mitochondrial protein has affinity to bind with eosin. Therefore, Hürthle cells are also called oxyphilic cells. Hürthle cell lesions in the thyroid are composed of cells with this classic histology, but not all oncocytic cells in the thyroid are true Hürthle cells [5]. Cells with less or incomplete eosinophilic, granular appearance can observed, at least focally, in any thyroid lesions, such as autoimmune thyroiditis, nodular goiter, aging, and irradiated thyroids. These oncocytic, non-Hürthle cells are called “oncocytic metaplasia”. Although immunohistochemistry using anti-mitochondrial markers can detect focal oncocytic metaplasia [6], there are no quantitative measurements to precisely distinguish between oncocytic metaplasia and non-oncocytic cells. True Hürthle cells have a cytoplasm filled with mitochondria with complete loss of cell polarity, while metaplastic cells do not have a complete loss of cell polarity and have fewer mitochondria at the ultrastructural level. Hürthle cell “tumors” are defined as oncocytic cells consisting of more than 75% of the tumor cells [7]. The oncocytic transformation itself is not related to tumor development and progression [6,8], but the number of mutations in mitochondrial DNA/nuclear mutation ratio seems to be higher [8]. The cellular origin of HCC, as well as other thyroid cancer types, is still not completely understood. Thyroid cancer stem cell (CSC) model is one hypothesis to explain thyroid carcinogenesis. To identify thyroid CSCs, various research with CD133 [9,10], CD44 [10,11], multi-drug resistance (MDR1) [12], ATP binding cassette subfamily G member 2 (ABCG2) [12,13], multi-drug associated protein 1 (MRP1) [13], aldehyde dehydrogenase(ALDH) [14], as well as sphere formation assay [15], and side population assay [14] have been carried out. These studies focused on differentiated and undifferentiated thyroid carcinomas, not on HCC, and no common CSC markers have been reported to date. Further studies on the isolation and characterization of CSCs in HCC will improve knowledge regarding HCC initiation.

Hürthle cell tumors used to be classified as a variant of follicular thyroid tumors. Hürthle cell tumors with capsular and/or vascular invasion, lymph nodes metastasis, or distant metastasis are HCC.

## 3. Clinical Features

### 3.1. Demographic Features

HCC is more often observed in females, and the reported female to male ratio of HCC is 1.6–4.8:1 [4,16,17,18]. The affected age range is 54–62 years old [4,17,19,20,21,22]. In a retrospective study, tumor size, frequencies of extrathyroidal extension, lymph node metastasis, and distant metastasis, were similar between HCC and non-oncocytic follicular thyroid carcinomas [16]. To date, no correlation with radiation exposure has been reported [23]. 

### 3.2. Laboratory Tests

Thyroglobulin production varies by case. Both high thyroglobulin production (>500 ng/dL) [24,25,26], and limited production [5], which reflects less active follicular cells, have been reported. An increased thyroglobulin level is a biomarker for recurrence after thyroidectomy; however, some recurrent HCC cases have undetectable thyroglobulin levels. Clinical follow-up by thyroglobulin levels should be decided depending on the case.

### 3.3. Ultrasounds

Preoperative diagnosis by ultrasound is challenging. In ultrasounds, HCC shows a range of sonographic appearances from predominantly hypoechoic to hyperechoic lesions, and no preoperative features can differentiate HCC from adenoma [18,27]. Several attempts have been made to predict HCC before the operation. Ito et al. classified thyroid nodules into classes 1 to 5 with intermediate steps of 0.5 for classes 2 to 5, called ultrasound classes (USC) [28]. USC2 is defined as having a round and cystic nodule/isoechoic solid nodule—an adenomatous nodule or follicular adenoma. USC3 is defined as a round, hypoechoic solid nodule—a follicular adenoma, adenomatous nodule, or possibly carcinoma. USC4 is a solid nodule with an irregular border or the presence of psammoma calcifications—a carcinoma. Although USC is not related to the incidence of HCC, among patients diagnosed with oncocytic cell tumors by fine-needle aspiration cytology (FNAC), such patients are more likely to show malignancy when USC is 3 or greater. 

### 3.4. Clinical Indicators

Indicators of malignancy among Hürthle cell tumors include male [29], tumor size >4 cm [18,23,26,29], US class ≥ 3 [26], older age (HCC 51.8 years old vs. Hürthle cell adenoma 43.1) [23]. Subsequently, surgical indications of Hürthle cell tumors are reported as USC ≥ 3, tumor size >4 cm, and thyroglobulin >500 ng/dL (with negative anti-thyroglobulin-antibody) [26]. 

## 4. Treatment

Either near total or total thyroidectomy or unilateral thyroidectomy is initially recommended for patients with HCC > 1 cm and <4 cm without extrathyroidal extension, and without lymph node metastasis [30]. Thyroid lobectomy alone is sufficient treatment for small, unifocal, intrathyroidal carcinomas in the absence of prior head and neck radiation, familial thyroid carcinoma, or clinically detectable cervical nodal metastases [30]. Total thyroidectomy is recommended for Hürthle cell neoplasms larger than 4 cm [31]. Whether total thyroidectomy as the primary procedure should be applied is controversial. While several studies showed more favorable outcome [17,29,31,32,33,34], the Surveillance, Epidemiology and End Results (SEER) in its large scale database, no significance difference in survival was observed between patients treated with total thyroidectomy and those treated with partial thyroidectomy [22]. Extensive surgery, external beam radiation, or chemotherapy did not confer a survival benefit [23].

The role of radioactive iodine (RAI) is still debated [16]. Guidelines regarding indications to use RAI for HCC are inconsistent, and RAI is not prevailing for HCC patients [35]. Jillard et al. suggested that RAI should be advocated for HCC patients with tumors > 2  cm and those with nodal and distant metastatic disease, as it improves survival [35]. Radioactive iodine therapy may prompt a survival benefit as an adjuvant ablation therapy, but only for those who do not have a residual disease [23]. Some teams may choose total thyroidectomy and RAI therapy. However, functionally, HCC shows decreased iodine uptake [25], resulting in lower responsiveness to RAI treatment [25]. 

## 5. Pathological Features

### 5.1. Cytological Features and Differential Diagnosis 

**Figure 1:**
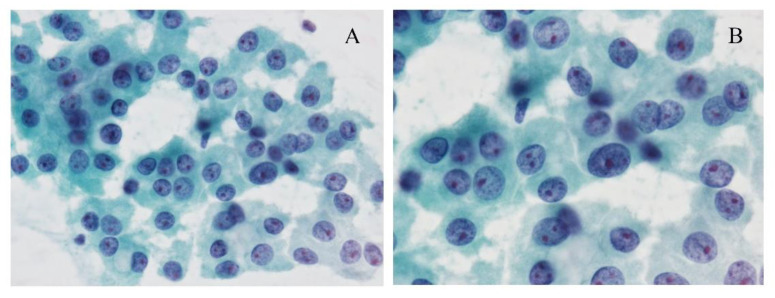
Cytological images of the Hürthle cell carcinoma (HCC). (**A**) The Papanicolaou-stained smear shows a monotonous cells oncocytic cells with prominent nucleoli arranged in loosely cohesive clusters. Papanicolaou stain, original magnification ×200. (**B**) The cells have prominent nucleoli, bland chromatin, and granular cytoplasm. Papanicolaou stain, original magnification ×600.

Using fine needle aspiration cytology (FNAC), clusters of the monotonous follicular cells with large eosinophilic, granular cytoplasm are observed, and usually, hypercellularity is noted (Figure 1). The cells often show irregular nuclear outlines, prominent nucleoli, and bland chromatin. The tumor cells appear scarcely accompanied by colloid, and/or inflammation.

Oncocytic cells are also observed in nodular goiter, chronic thyroiditis, and oncocytic variants of papillary thyroid carcinoma and oncocytic variant of medullary thyroid carcinoma (OV-MTC). Therefore, the presence of these cells should not engender a diagnosis of Hürthle cell lesion [5]. In chronic thyroiditis and nodular goiter, isolated or small cohesive clusters of oncocytic cells are common, representing follicular cells with oncocytic metaplasia. In addition, in nodular goiter, follicular cells with abundant colloids are observed, and the follicles are not uniform. In chronic thyroiditis, lymphoplasmacytic infiltrations are frequently observed. However, when a large irregular sheet of oncocytic cells dominantly appear, differentiating between chronic thyroiditis and Hürthle cell tumor may be difficult. Indicators of true Hürthle cell tumors are (1) microfollicular architecture, (2) absence of colloid, (3) absence of inflammation, and (4) presence of transgressing blood vessels [36,37]. 

The tumor cells of oncocytic variants of papillary thyroid carcinomas show fine granular cytoplasms and elongated nuclei, and a nuclear groove. Intracytoplasmic inclusion can be sparse in some cases. The cytological features of papillary fronds and monolayered sheets suggest an oncocytic variant of papillary thyroid carcinoma rather than Hürthle cell tumors [38,39]. In the FNAC sample of the OV-MTC, amyloid deposit supports the diagnosis, but is not always observed [40]. OV-MTC cells show polygonal, plasmacytoid characteristics with eccentrically placed round nuclei with “salt and pepper” chromatin [41]. Multi-vacuolization resembling histiocytes and loose granularity is observed in OV-MTC. In contrast, granularity in HCC is dense and firm [42]. For accurate diagnosis, immunohistochemistry using calcitonin and thyroglobulin in combination [43], as well as consideration of family history of the patient and laboratory data are needed.

Cytologic features such as small cell dysplasia (bland nuclei, cell diameter less than twice the nuclear diameter), large cell dysplasia (cell diameter at least twice the nuclear diameter, with prominent nucleoli and irregular nuclear outlines), nuclear crowding, and dyshesion have been proposed as a diagnostic clue for HCC [44]. These criteria were later shown by others to be significant features in favor of HCC over adenoma and other benign lesions [45]. However, distinction of HCC from Hürthle cell adenoma by FNAC still remains to be challenging due to the lack of other supporting data [46]. In the daily practice using Bethesda reporting system for thyroid cytology (TBSRTC) [47], a Hürthle cell neoplasm is typically classified as atypia of undetermined significance or follicular lesion of undetermined significance (AUS/FLUS, Bethesda III) or benign (Bethesda II). The appearance of Hürthle cell is encouraged to comment. The presence of Hürthle cells itself does not increase the risk of malignancy [48], but > 75% Hürthle cells in a benign/Bethesda II aspirate may pose an increased risk of malignancy [49]. 

### 5.2. Macroscopic Features

**Figure 2:**
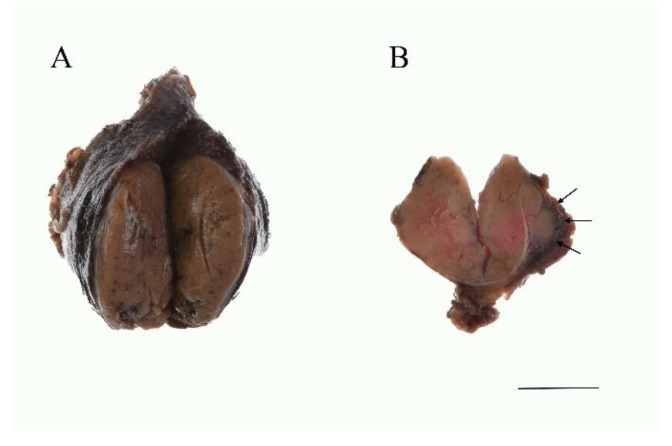
Gross findings of the HCC. (**A**) The tumor is mahogany brown color. Several foci of hemorrhage are noted. (**B**) Widely invasive HCC. Macroscopically, capsular invasion is evident (arrows). Scale bar indicates 2 cm.

Macroscopically, HCC shows a mahogany brown appearance (Figure 2), which is due to air contact [25]. Necrosis and hemorrhaging may be observed due to its degenerative nature. Sometimes calcification is observed and is more often found in HCC than in Hürthle cell adenoma. Calcification is a result of a physiochemical reaction to altered thyroglobulin within the colloid of the tumor follicles. 

### 5.3. Histological Features and Differential Diagnosis at Histology

**Figure 3:**
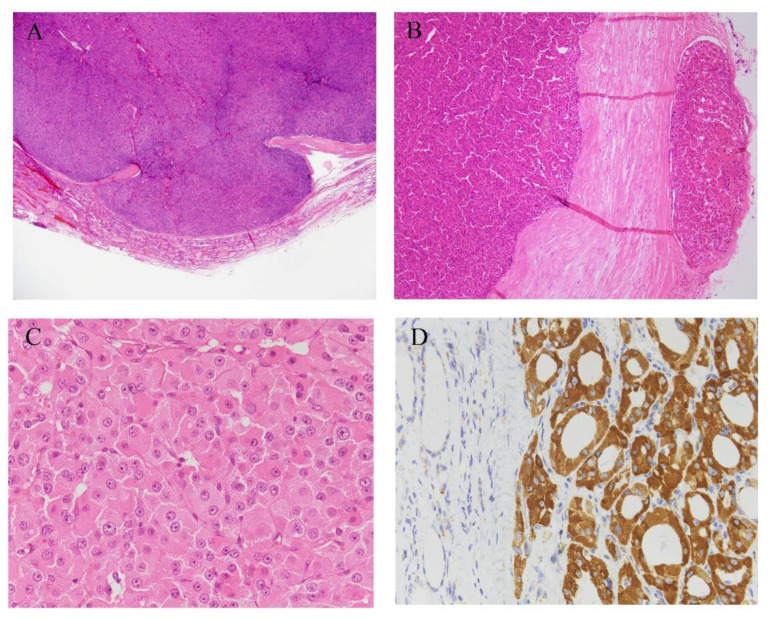
Histological images of HCCs. (**A**) HCC is diagnosed in cases where a Hürthle cell tumor shows capsular invasion, and/or (**B**) vascular invasion. (**C**) HCC is composed of polygonal large cells with abundant granular and acidophilic cytoplasm. HCC cells have large nuclei with prominent nucleoli. (**D**) Tumor cells are diffuse and strongly positive for anti-mitochondria antibodies. Hematoxylin and eosin stain (**A–C**), immunohistochemistry of anti-mitochondrial antibody (**D**). Original magnification, ×1.25 (**A**), ×40 (**B**), ×100 (**D**), and ×400 (**C**).

In cases where the Hürthle cell tumor showed capsular invasion and/or vascular invasion, HCC is diagnosed (Figure 2B and Figure 3A,B). Based on its histology, the tumor is subcategorized as either minimally invasive or widely invasive. Minimally invasive HCC refers to encapsulated tumors with microscopically identifiable foci with capsular or vascular invasion less than four foci. Widely invasive HCC shows extensive capsular invasion and/or vascular invasion of four foci or greater. 

HCC often causes infarction after FNAC. This may make it difficult to observe the capsular invasions. Mitochondrial DNA common deletion is reported to be less frequent in infarcted Hürthle cell tumors than in non-infarcted Hürthle cell tumors. Still, the mechanism leading to infarction is unknown [50]. For clear identification of capsular invasion, clinical information such as where the tumor cells were aspirated by FNAC is considered important. Widely invasive HCC often shows capsular invasion in the form of compacted nodules, lacking stromal desmoplastic reaction. In some cases, determining whether there is a capsule is difficult.

HCC is composed of pleomorphic or polygonal large cells with abundant, granular, and acidophilic cytoplasm and large nuclei with prominent nucleoli (Figure 3C) [51]. It often shows follicular, trabecular, and/or solid architecture, and rarely shows a predominantly papillary pattern [25]. A pure follicular pattern is less common in HCC [1], but when such a follicular pattern is seen, the tumor accompanies fibrous bands between nests.

Cytoplasmic granularity in Hürthle cells is due to the presence of numerous mitochondria (Figure 3D). The amount and morphological characteristics of the mitochondria vary greatly from case to case [52]. However, according to the diagnostic criteria, more than 75% of oxyphilic cells must be observed for diagnosis. The high prevalence of Hürthle cell changing into thyroid lesions may reflect high oxidative stress and reactive oxygen species production in thyroid cells during normal iodine and thyroid hormone metabolism [53,54]. These high reactive oxygen species levels can result in mutagenic genetic events in mitochondrial DNA, leading to mitochondrial dysfunction [7,55]. Abnormality of mitochondria leads to defects in the energy production process, and a compensatory mechanism for these defects is considered to come into play [7]. This results in increased proliferation of numerous mitochondria, which is observed as oncocytic cytoplasm. Over time, as the number of the mitochondria increases, Hürthle cell appearance is expressed [51], and this is a continuous process rather than a “black-and white phenomenon” [7]. In cases of HCC, necrosis frequently appears either spontaneously or after fine-needle aspiration. This observation is reported to be as a consequence of mitochondrial abnormalities [56].

Thyroid lesions containing oncocytic cells are nodular goiter, oncocytic variants of papillary carcinoma and medullary carcinoma. Oncocytic variants of medullary thyroid carcinoma (MTC) are rare, showing extensive mitochondrial hyperplasia, prominent nucleoli, well-defined cytoplasm, and polygonal cells, which is in contrast with the spindle-shaped cells of conventional MTC. Although MTC is derived from C cells (parafollicular cells), not follicular cells, oncocytic variant MTC may be difficult to histologically differentiate. MTC is often accompanied by amyloid deposits, and this aids in diagnosis. However, amyloid deposits sometimes occur as a result of systemic or secondary amyloidosis [57]. HCC with neuroendocrine differentiation [58] or thyroid tumors in patients with basal high calcitonin levels should be diagnosed with caution. For the appropriate diagnosis, consideration of molecular testing, as well as family history and serum calcitonin levels, is essential [59]. Oncocytic parathyroid adenoma and adenocarcinoma also require differential diagnosis. Neoplastic lesions showing extensive invasion without hormone production are especially difficult to differentiate. Immunohistochemically, parathyroid tissue tests positive for chromogranin A, GATA3, while thyroid tissue tests positive for TTF-1, PAX8. However, in the hormone non-producing tumors, neither calcitonin nor PTH is clearly expressed. Tumor location with images and invasive patterns can be diagnostic clues in these cases. 

Immunohistochemistry is not mandatory for the diagnosis of HCC, except in cases to distinguish MTC or parathyroid tumors, and a specific diagnostic marker for HCC has not yet been obtained. HCC cells test positive for thyroid transcription factor-1 (TTF-1) and thyroglobulin, and negative for calcitonin and parathyroid hormone (PTH). Thyroglobulin may show perinuclear positivity. According to the literature, genes associated with Retinoid-interferon-induced Mortality-19 (GRIM-19) [60], p53 [56], and Cyclin D [61,62] can be immunohistochemically detected. 

### 5.4. Ultrastructure

**Figure 4:**
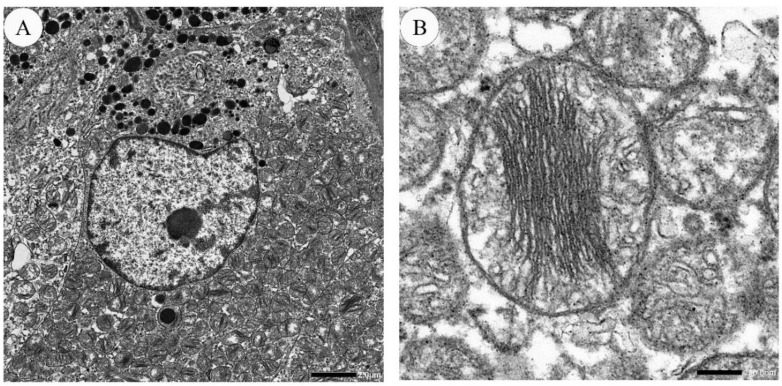
Electron microscopic images of HCC. (**A**) An HCC cell contains an irregular and notched nucleus. Cytoplasm is filled with numerous mitochondria. The bar indicates 2.0 micrometers. (**B**) Mitochondria display packed stacking cristae, arranged in the center. The bar indicates 200.0 nanometers.

Using transmission electron microscopy, HCC cells are observed to have large, irregular nuclei with a deep notch [63]. Nuclei contain one to several nucleoli, and the cytoplasm is packed with several thousand mitochondria (Figure 4). A full-brown oncocytic cell is estimated to contain 4000 to 5000 mitochondria [64]. These mitochondria display two types of morphology. One is larger mitochondria with stacking “shelf-like” cristae [63,65]. The other is smaller and oval, and short cristae are peripherally arranged [63]. Other structural abnormalities are bundles of dense filaments, filamentous inclusions, and round electron-dense bodies in the matrix [5,51,66]. The amount and morphological characteristics of the mitochondria vary greatly from case to case [51,52]. The surface of HCC cells is irregular. 

## 6. Molecular Changes

The genetic profile of HCC differs from papillary thyroid carcinoma and follicular thyroid carcinoma. Molecular pathways that differentiate Hürthle cell adenoma from HCC with wide invasiveness included the PIK3CA-Akt-mTOR and Wnt, providing rationale toward new targets of this type of malignancy [67]. A significant difference in oncogene expression between follicular thyroid carcinoma (FTC) and HCC suggests that despite having a common origin in the follicular cell, follicular thyroid carcinoma and HCC should be considered as two separate entities [68]. HCC shows changes in both nuclear DNA and mitochondrial DNA (mtDNA). Molecular changes in nuclear DNA involve several signaling pathways and mitochondrial functions. Mitochondrial genetic changes involve interaction between mutations in the mitochondrial and nuclear genomes [55]. 

### 6.1. Genetic Change in Genes Involved in RAS/RAF/MAPK and PIK3/Akt/mTOR Pathways

Ganly et al. identified genetic mutations associated with RAS/RAF/MAPK and PIK3/Akt/mTOR pathways in HCCs [67,69]. These genes included PTEN, PIK3, PTEN, TSC1, TSC2, and RAS family. PTEN was observed in about 5% of cases overall [69,70] and in 25% of TP53 mutation positive-cases [2]. RAS family mutations—NRAS, KRAS, and HRAS—are identified in about 45% of follicular tumors. In HCC, the incidence of RAS mutations is less than that of conventional follicular tumors, which is about 10–15% [67,69,71,72]. BRAF mutations are the predominant genetic changes in papillary thyroid carcinoma but the incidence of BRAF mutation in HCC is not common [25].

### 6.2. Genetic Rearrangements

Along with the point mutations, chromosomal rearrangements are also common mutational mechanism in thyroid cancers [73]. However, genetic rearrangements of PAX8/PPAR, RET/PTC, EML4/ALK, and ETV6/NTRK3 are infrequently observed in HCC [25,72,74]. 

### 6.3. TP53

This mutation is known to occur with increasing frequency in dedifferentiated thyroid tumors rather than well-differentiated tumors. TP53 mutations were found at a high allelic frequency [70]. The TP53 mutation was found in 22% cases overall [70], and in 42% [2] of HCC cases. All the mutations are in the DNA binding domain [70]. This mutation may occur in tumors that are prone to dedifferentiation and can therefore be used as a diagnostic and prognostic marker in oncocytic follicular tumors. 

### 6.4. TERT Promoter Mutations

TERT promoter mutations are associated with unfavorable prognosis in various cancers. Major TERT promoter mutations are C228T and C250T. TERT promoter mutations are found mainly in widely invasive HCCs (32%), and only in 5% of minimally invasive HCCs [4,72]. In a recent study, some thyroid tumor showed increased TERT mRNA expression even in the absence of *TERT* promoter mutations, which have a significantly high recurrence rate. [75]. In HCCs, increased TERT mRNA expression has not been observed [4]. 

### 6.5. Tumor Mutational Burden and Global Copy Number Diversity

Tumor mutational burden (TMB) refers to the number of mutations that exist within a tumor [76]. HCCs contain an average of 2.6 mutations/Mb, which was six-fold greater than that reported in The Cancer Genome Atlas (TCGA) of PTC (0.41 mutations/Mb) [72]. High TMB has been associated with responses to immune checkpoint inhibitors in several tumors, such as non-small cell lung cancer, and can be a promising biomarker. Still, its cutoff and clinical relevance for thyroid tumors have not yet been elucidated. 

Regions of chromosomal gain and loss are observed in HCCs. Large regions of amplification are observed on chromosomes 5, 7, 12, and 17. These chromosomal amplifications potentially lead to activation of RAS-RAF-MEK and PI3K-Akt-mTOR pathways [67]. Chromosome 7 and 12 potentially activate the RAS-RAF-MEK pathway. Chromosomal regions of gain and loss in HCC differ from papillary thyroid carcinoma and follicular thyroid carcinoma [67]. Through amplification of chromosome 7, BRAF was found to be significantly overexpressed in HCC, which is involved in the mTOR pathway. According to clustering analysis of the copy number data, three groups of Hürthle cell tumors were observed—deletions predominant type, amplification predominant type, and mixed type. Among these, the mixed type was mostly attributed in aggressive HCC [67]. Loss of heterogeneity (LOH) has been reported in HCC [72,77]. In two studies, LOH is associated with more aggressive diseases. However, the reason why LOH leads to a worsened course is unknown. 

### 6.6. Mutations in Mitochondrial DNA and Related Genes

The mitochondrial DNA (mtDNA) is small, and incorporates a sequence of 16,569 bp, encoding 13 essential components in cellular energy production [51]. Mitochondrial DNA (mtDNA) is more susceptible than nuclear DNA to mutagen-induced damage. As in other types of cancers [78,79], alterations in mtDNA in HCC have been revealed. So-called “common deletion” is the most common mitochondrial alteration. It is a large-scale deletion of 4977 base pairs (bp) of mtDNA residing between two 13-bp direct repeats in the mtDNA sequence at nucleotide positions 13,447–13,459 and 8470–8484. Hürthle cell tumor displayed high percentage of the common deletion. Common deletion was significantly higher in Hürthle cell tumors than in non-Hürthle cell tumors, and significantly higher HCC [80]. Disruptive mutations in mitochondrial DNA are found in complex I subunit genes in Hürthle cell tumors. Usually, mutations of mtDNA are heteroplasmic, but homoplasmy in the Hürthle cell tumors is frequently observed. Gasparre et al. sequenced the entire mtDNA in thyroid oncocytic tumors and controls [81], and 26% of the analyzed thyroid oncocytic tumors showed disruptive mutations (nonsense or frameshift). This was observed in complex I subunit genes, and the association between these mutations and the oncocytic phenotype was statistically significant [81]. Attributing a causal role to single mitochondrial mutation is difficult, and a more-than-one-hit hypothesis is more plausible. Mitochondrial mutations may play a part as one of many factors leading to tumor development [81]. 

The genes associated with Retinoid-interferon-induced Mortality-19 (GRIM-19), one of the IFN/RA inducible GRIM products, is a 552-base pair cDNA mapped to human chromosome 19p13.2 [82], which encodes a 16-kDa protein. It was originally identified as a potential tumor suppressor associated with growth inhibition or cell apoptosis. Subsequently, it was identified as an essential subunit of the mitochondrial respiratory chain complex I [83,84]. It also represses STAT3 and serves as a negative regulator of cell growth [82,85]. In the colorectal cancer cell lines, GRIM-19 was shown to inhibit hypoxia-induced autophagy through inactivation HIF-1α/STAT3 dependent gene transcription, and suppress hypoxia-triggered invasion and epithelial-mesenchymal transition (EMT) [86]. Somatic missense mutations in GRIM19 has been found in approximately 11% of HCCs [87]. GRIM-19 may potentially aid as a cytological marker of malignancy in Hürthle cell tumors [60].

Variations in the inner mitochondrial membrane transporter have been also reported. Mitochondrial protein is mostly encoded by the nuclear genome. These are imported from cytosol into mitochondria via translocator, translocase of inner mitochondrial membrane 44 homolog (TRMM44) [88]. Variations in the inner mitochondrial membrane transporter TIMM44 have been observed in patients with oncocytic thyroid tumors. Details of how these variation cause or affect HCC is still not known. 

### 6.7. Other Molecular Changes

Differences between of minimally invasive HCC and widely invasive HCC could be demonstrated by gene expression analysis. Among these differences, beta-catenin (CTNNB1) was a significant gene set that was enriched [67]. In widely invasive HCC, beta-catenin is involved in processes regulating vascular invasion [67]. 

Sheu et al. showed that oncocytic thyroid tumors, including adenoma and carcinoma, showed a statistically significant increase in C-allele frequency of GNB3 C825T polymorphism of the G protein beta3-subunit compared to all non-oncocytic tumors [89]. GNB3 C825T has been reported to contribute to increased risk of cancer, especially thyroid carcinoma [90]. This polymorphism may thus be a (co)factor favoring the development of oncocytic thyroid tumors, but the biological mechanism remains obscure. Whether this is somehow related to the mitochondrial DNA changes seen in oncocytic thyroid tumors also remains to be seen [51].

Among genetic changes, HCCs can be divided into three groups according to the combination of TERT promoter mutation, widespread LOH, and whole-chromosome duplication of chromosome 7 assigns [72]. The first group shows TERT alterations, major LOH, and whole-chromosome duplication (WCD) of chromosome 7 and is observed in widely invasive HCC. The second group shows major LOH and WCD of chromosome 7, without TERT alteration. The third group does not show these genetic changes, which are observed in minimally invasive and nonrecurrent HCCs. Other molecular changes need further assessment of their biological meanings. 

## 7. Prognosis and Prognostic Factors

Overall recurrence rates and average time to relapse are 12–33% and 90.74 months [17,32]. Average time without symptoms of disease is 222.4 months [17]. The 5-year and 10-year cancer-specific survival rates are 85–95.4 and 92.6%, respectively [4,17,22]. One in four patients at presentation is M1 stage [4], and one in four patients develops metastatic disease, with a median time of 50 months [32].

The five-year cumulative probability of recurrence or mortality among patients with TNM stage I–II among female and male patients were 0% and 17%, respectively. Among patients with TNM stage III–IV, the five-year cumulative probability of recurrence or mortality for female and male patients were 74% and 91%, respectively [4].

HCC has been thought to lead to a worse prognosis than that for non-oncocytic tumors. Prognosis differs between minimally and widely invasive HCCs. As long as the tumor is minimally invasive and non-angioinvasive, prognosis is excellent. No recurrence was observed only among patients with minimally invasive HCCs [4]. If widely invasive HCC is observed in males, older age (>45) with more than four foci of angioinvasion, larger than 4 cm, and/or TNM stage III–IV, prognosis is poor [4,31]. Widely invasive HCCs are significantly associated with the male gender and clinical recurrence or mortality.

## 8. Conclusions

HCC has been established as a separate entry in the current WHO classification. Morphologically, HCC demonstrates a distinct feature which is caused by mitochondrial accumulation. HCC can lead to worse prognosis in cases where the tumor is widely invasive, in male patients, older age, more than four foci of angioinvasion, tumor larger than 4 cm, and/or TNM stage III–IV. HCC shows changes in both nuclear DNA and mtDNA involving signaling pathways and mitochondrial functions. Further clarification of the biological meaning of the molecular features is needed for molecular-based, personalized medicine.

## Data Availability

Data available on request due to restrictions eg privacy or ethical.
